# Relationship between depth-wise refractive index and biomechanical properties of human articular cartilage

**DOI:** 10.1117/1.JBO.29.9.095003

**Published:** 2024-09-20

**Authors:** Bilour Khan, Ervin Nippolainen, Fatemeh Shahini, Nonappa, Alexey Popov, Juha Töyräs, Isaac O. Afara

**Affiliations:** a University of Eastern Finland, Department of Technical Physics, Kuopio, Finland; b Tampere University, Faculty of Engineering and Natural Sciences, Tampere, Finland; c VTT Technical Research Center of Finland, Oulu, Finland; d Kuopio University Hospital, Science Service Center, Kuopio, Finland; e The University of Queensland, School of Electrical Engineering and Computer Science, Brisbane, Australia

**Keywords:** articular cartilage, refractive index, biomechanical properties, optical properties, tissue integrity, collagen fibril orientation

## Abstract

**Significance:**

Optical properties of biological tissues, such as refractive index (RI), are fundamental properties, intrinsically linked to the tissue's composition and structure. We hypothesize that, as the RI and the functional properties of articular cartilage (AC) are dependent on the tissue’s structure and composition, the RI of AC is related to its biomechanical properties.

**Aim:**

This study aims to investigate the relationship between RI of human AC and its biomechanical properties.

**Approach:**

Human cartilage samples (n=22) were extracted from the right knee joint of three cadaver donors (one female, aged 47 years, and two males, aged 64 and 68 years) obtained from a commercial biobank (Science Care, Phoenix, Arizona, United States). The samples were initially subjected to mechanical indentation testing to determine elastic [equilibrium modulus (EM) and instantaneous modulus (IM)] and dynamic [dynamic modulus (DM)] viscoelastic properties. An Abbemat 3200 automatic one-wavelength refractometer operating at 600 nm was used to measure the RI of the extracted sections. Similarly, Spearman’s and Pearson’s correlation coefficients were employed for non-normal and normal datasets, respectively, to determine the correlation between the depth-wise RI and biomechanical properties of the cartilage samples as a function of the collagen fibril orientation.

**Results:**

A positive correlation with statistically significant relations (p−values<0.05) was observed between the RI and the biomechanical properties (EM, IM, and DM) along the tissue depth for each zone, e.g., superficial, middle, and deep zones. Likewise, a lower positive correlation with statistically significant relations (p−values<0.05) was also observed for collagen fibril orientation of all zones with the biomechanical properties.

**Conclusions:**

The results indicate that, although the RI exhibits different levels of correlation with different biomechanical properties, the relationship varies as a function of the tissue depth. This knowledge paves the way for optically monitoring changes in AC biomechanical properties nondestructively via changes in the RI. Thus, the RI could be a potential biomarker for assessing the mechanical competency of AC, particularly in degenerative diseases, such as osteoarthritis.

## 1 Introduction

Articular cartilage (AC) is a specialized connective tissue covering the ends of bones in diarthrodial joints, such as the knee, hip, and shoulders.^1^ The primary function of AC is the provision of frictionless articulation and transmission of physical loading to the underlying subchondral bone without leading to high stresses.^2^ Mature AC has limited repair capability due to the lack of vasculature and nerves; thus, it is susceptible to degenerative changes driven by age or injury. The extracellular matrix (ECM) of AC mainly comprises ∼5% to 10% proteoglycan (PG) macromolecules, 10% to 20% collagen fibril meshwork, and around 60% to 80% water.^3^

The structure and composition of AC vary along the tissue depth.^4^^,^^5^ Structurally, AC can be characterized into four distinctive zones based on collagen fibril orientation. These zones are the superficial zone (SZ), middle zone (MZ), deep zone (DZ,) and calcified zone (CZ). The SZ comprises ∼0% to 10% of AC overall thickness,^6^ with collagen fibers aligned parallel to the articular surface of the tissue. The SZ is in direct contact with synovial fluid present in the joint space and protects the ECM from shear stresses. The transitional zone or MZ covers ∼10% to 30% of AC thickness and plays a key role in load transmission. Collagen fibers in the MZ exhibit random orientation. The DZ covers ∼70% to 90% of the ECM^6^ and interfaces with CZ. The DZ provides the highest resistance to compressive loading^2^ and has collagen fibers oriented perpendicular to the cartilage–bone interface. The water content decreases, the PG content increases, and the density of the collagen fibril network increases from the SZ to the DZ.^5^^,^^7^^,^^8^ Furthermore, in the SZ, chondrocytes are flattened, whereas they exhibit a spherical shape in the MZ and DZ. In the DZ, chondrocytes have a columnar cluster arrangement with vertical orientation, parallel to the collagen fibers.^5^ The SZ holds a relatively higher number of chondrocytes compared with the MZ and DZ.^5^

Compositionally, PG macromolecules are responsible for cartilage compressive properties, whereas collagen fibrils contribute more to the dynamic and tensile properties.^9^^,^^10^ Alteration of the depth-wise profile of the AC matrix constituents often results in progressive degeneration, leading to osteoarthritis (OA), one of the most prevalent degenerative joint diseases, with a global prevalence estimated to be up to 28% in adults more than 60 years old.^11^ OA is a multifaceted joint disease primarily linked with disruption of collagen fibers network,^12^ loss of PG content, increased matrix water content, and subsequent compromise of the mechanical function of the tissue.^2^

The refractive index (RI) is one of the primary optical properties that characterizes the manner in which light interacts with a material as it traverses through it.^13^ The RI of biological tissues is a key parameter that contributes to the understanding of light propagation through the tissue, potentially providing diagnostic information on tissue integrity^2^ and providing insight into their molecular composition and structural integrity. Previous studies have highlighted the importance of polarized light interactions in characterizing these optical properties.^14^

Recent studies^13^^,^^15^^,^^16^ have reported that both absorption and scattering of the incoming light, combined with the RI, play critical roles in understanding the interaction of light in biological tissues. For example, tumor development in biological tissues is often characterized by significant alteration in the morphology and density of cells in the pathological tissue; these structural changes impact the light scattering patterns, which can subsequently be used as a biomarker for tissue characterization (e.g., healthy versus cancerous).^15^ In addition, studies have presented key findings related to the variation of RI values across samples from human liver, kidney, myocardium, and skin,^16^ suggesting the RI as a potential optical property for tissue characterization. The RI has shown significant potential for the characterization and diagnostic assessment of tissue pathologies.^13^ As AC is a highly anisotropic, optically turbid, and inhomogeneous tissue,^17^ its scattering nature strongly influences the propagation of light, which is further determined by the tissue composition, integrity, and structure along the optical path.^18^

Biomechanical properties of AC originate from the multi-phasic heterogeneous structure of the ECM. The constituents of the ECM and their interactions determine the non-linear mechanical response of AC.^19^^,^^20^ PGs are the primary regulator of AC mechanical behavior under the scenario in which the interstitial fluid flow has seized.^21^ Furthermore, both the collagen fiber meshwork and the interstitial fluid of the ECM control the mechanical behavior of AC during cyclic and transient loads.^22^ OA is known to have significant impacts on the AC structure and function during the early disease stages. For example, PG loss is progressive and results in the reduction of cartilage load-bearing capacity in equilibrium.^23^ Subsequently, disease progression leads to surface fibrillation due to collagen network damage, and a portion of the tissue swelling–related pretension of the fibers is compromised. Degenerative changes in the structure and composition of the ECM lead to an increase in the hydraulic permeability of the tissue, which decreases the interstitial fluid pressurization and weakens the transient response of cartilage.^24^^–^^26^ However, scientific studies on the effect of alteration of human AC biomechanical properties on its optical properties are scarce. As far as we know, no study has investigated the relationship between the human AC RI and its biomechanical properties.

In this study, we hypothesize that there is a relationship between the depth-wise RI of human AC and its biomechanical properties. This hypothesis is based on our initial findings that the RI of human AC varies with its ECM structure and composition.^27^ Furthermore, the scientific basis for the hypothesis is supported by the depth-dependent variation of AC major constituents, e.g., collagen fibers, chondrocytes density and shape, water distribution, and PG content. Consequently, it is our position that the depth-dependent composition and structure of human AC will result in varying light–tissue interaction along the tissue depth, resulting in varying relationships between the biomechanical properties of the tissue and RI along the tissue depth. To test this hypothesis, the RIs of tissue sections extracted from different depths of human patellar articular cartilage samples were measured, and their relationship with the bulk tissue biomechanical properties was investigated.

## 2 Materials and Methods

### 2.1 Specimens Preparations

**Fig. 1:**
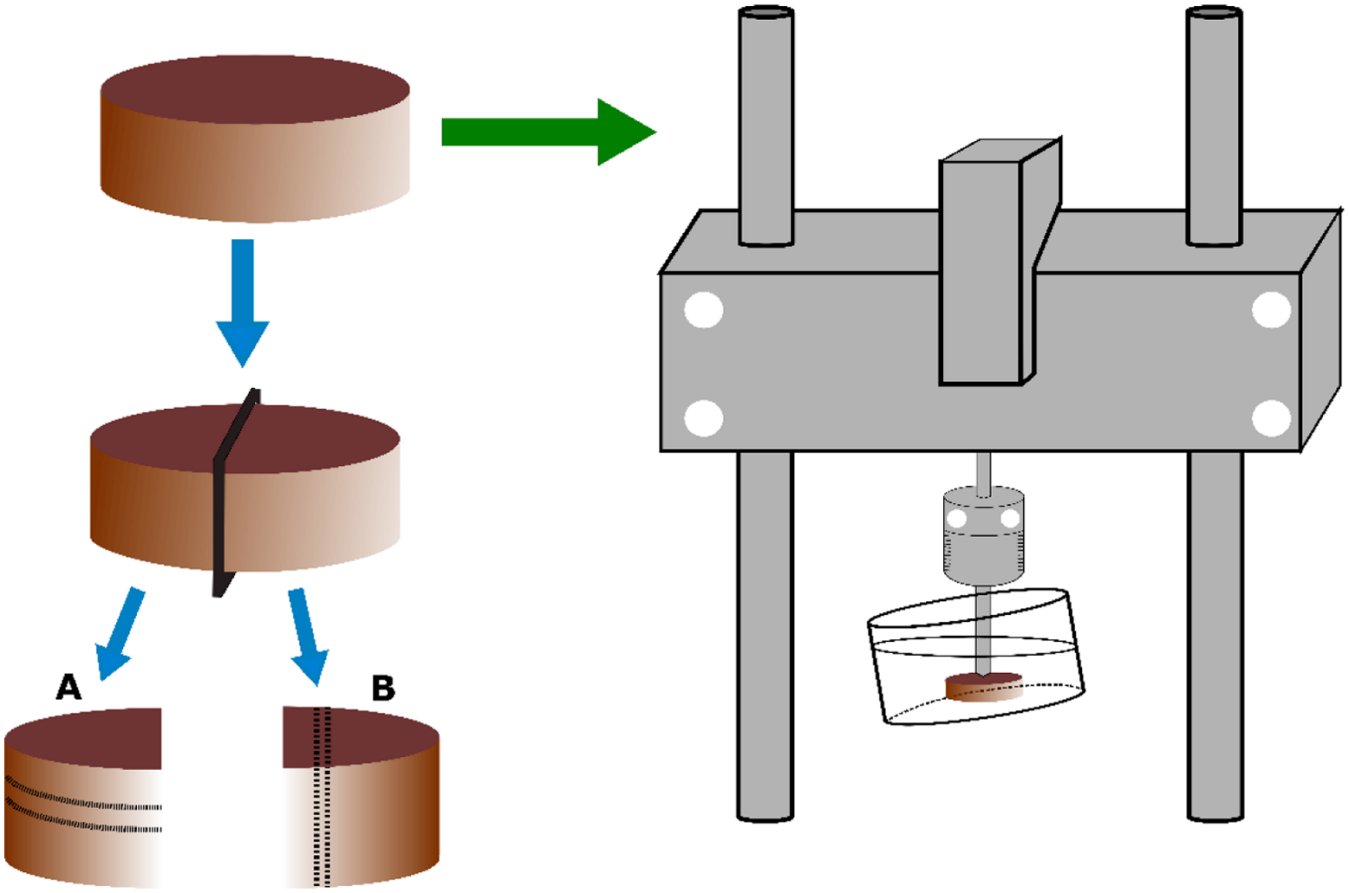
Schematic illustration of the experimental setup for sample preparation. Horizontal sections were extracted from specimen A for RI measurement, and vertical sections were extracted from specimen B for histological assessment and microscopy (PLM). Mechanical indentation testing was performed to measure elastic (EM and IM) and dynamic viscoelastic (DM) properties of the samples.

Human cartilage samples (n=22) were extracted from the right knee joint of three cadaver donors (one female, aged 47 years, and two males, aged 64 and 68 years) obtained from a commercial biobank (Science Care, Phoenix, Arizona, United States). Sample collection was approved by the Research Ethics Committee of the Northern Savo Hospital District (Kuopio University Hospital, Kuopio, Finland, #134//2015). The numbers of samples obtained from the three donors were eight, six, and eight. The samples were extracted from the central and anterior parts of the medial femur (n=5 and 3, respectively) and the central and anterior parts of the lateral tibia (n=6 and 4, respectively). The samples were extracted as cylindrical osteochondral plugs using a dental drill (diameter = 8 mm). The samples were initially subjected to mechanical indentation testing to determine the elastic [equilibrium modulus (EM) and instantaneous modulus (IM)] and dynamic [dynamic modulus (DM)] viscoelastic properties and then divided into two specimens: A and B as shown in Fig 1. Specimen A was subjected to RI measurement, whereas histological assessment was performed on specimen B (already reported in our previous study^27^). The thickness of the cartilage in specimen A was determined as the mean value of thickness measurement from the sides of the specimen, using an optical microscope (Zeiss, STEMI, SV8, Oberkochen, Germany).^28^

Specimen A was then immersed in phosphate-buffered saline (PBS) and stored at −20°C before undergoing the cryosectioning procedure. To measure the RI of different cartilage layers, tissue sections (200  μm) were extracted from depths corresponding to the different zones of the articular cartilage (SZ, MZ, and DZ) using a cryosection procedure. In summary, the bone end of each plug was flattened and adjusted to be parallel with the articular surface using a bandsaw. The flattened bone end was then attached to a disc using a fixative medium (optimal cutting temperature compound, Thermo Fisher Scientific Ltd., Runcorn, United Kingdom). The disc was then mounted in the cryostat chamber (Leica CM3050 S, Leica Biosystems, Wetzlar, Germany) for ∼5  min at −20°C to undergo rapid freezing, followed by placement on the microtome within the chamber. The microtome has a cutting range of 0.5 to 300  μm, with a precision orientation of tissue sections of ∼8  deg. Prior to sectioning, a disposable blade (MX35 Ultra, Thermo Fisher Scientific Ltd., Runcorn, United Kingdom) with a thickness of 0.24 mm and a cutting angle of 34 deg was set inside the knife holder of the cryostat chamber, and the first 50  μm of the articular surface was cut and discarded to create a uniformly flat surface for sectioning. The removal of this initial section of the articular surface is due to the naturally curvy surface of the tissue, thus ensuring the flatness of the subsequently extracted sections. After flattening the articular surface, 10 consecutive 200-μm-thick sections were extracted and mounted on glass slides (thickness = 1 mm, Menzel–Gläser frosted microscope slides, Thermo Fisher Scientific Oy, Vantaa, Finland) and then stored for RI measurement in a humid box at 4°C. In total, 220 sections were extracted for the analysis. It is important to mention that the biomechanical properties of articular cartilage are not significantly altered by the freeze-thaw cycle, as established in previous studies, including research conducted by our group.^29^ Therefore, performing mechanical measurements on fresh cartilage and subsequent optical measurements post-freezing is a valid and widely accepted approach.

Histological evaluation was performed on the B specimens. The samples were fixed and decalcified in a solution containing formaldehyde and ethylene diamine tetra acetic acid for 21 days, followed by processing and embedding in paraffin for sectioning.^30^ For the measurement of collagen fibril orientation, 5-μm histological sections were cut perpendicular to the articular surface. Likewise, the 3-μm histological sections were cut perpendicular to the articular surface and stained with Safranin O for digital densitometry and Mankin, and Osteoarthritis Research Society International (OARSI) scoring, being used in our previous study.^27^ Identification of cartilage zones was based on the depth-wise orientation of the collagen fibers derived from polarized light microscopy (PLM). Thus, the SZ, MZ, and DZ were defined as collagen fibril orientation of approximately 20 deg or less, >20 and <70  deg, and >70  deg, respectively. Based on the literature,^28^^,^^30^^,^^31^ the SZ, MZ, and DZ correspond to 0% to 10%, 10% to 30%, and 30% to 100% of the overall cartilage thickness, respectively. During the preparation and extraction of the samples, the tissue surface was kept hydrated at regular intervals with PBS containing protease inhibitors.^32^

### 2.2 Indentation Testing

To measure the mechanical properties of the cartilage, an indentation test with a uniform plan-ended indenter (1 mm diameter) was performed on the osteochondral samples (8 mm diameter), before splitting the sample into two. EM was determined using stepwise indentation stress relaxation tests and calculated from the slope of the linear least-squares fit to the equilibrium stress–strain points. The sample was mounted on a rigid sample holder followed by compression with the plane-ended indenter, with a pre-stress of 12.5 kPa followed by a four-step stress relation test.^33^ Each step consisted of 5% strain and 15 min of relaxation time. Based on a previous study, which reported an optically measured Poisson’s ratio for bovine tibial cartilage,^34^ the Poisson’s ratio at equilibrium was set to 0.3.^19^ Similarly, the IM of cartilage was calculated from each peak stress point. Thus, we can obtain data points for the IM as a function of the applied strain. Due to the high loading rate, the samples were assumed to be incompressible, and the Poisson’s ratio was set to 0.57.^35^ The obtained strain-dependent instantaneous moduli were corrected using Hayes equation,^36^ which takes into account the measurement geometry. The DM was determined from a dynamic sinusoidal test, conducted using 2% strain amplitude with frequencies of 0.005, 0.05, 0.1, 0.25, 0.5, 0.625, 0.833, and 1 Hz. The DM was defined as the ratio of stress and strain amplitudes in the dynamic sinusoidal test.^19^ During dynamic testing, the samples were assumed incompressible, having Poisson’s of 0.5.^35^

### 2.3 Refractive Index Measurement

An Abbemat 3200 automatic one-wavelength refractometer operating at 600 nm was used to measure the RI of the extracted sections from specimen A. The device is equipped with an automatic temperature controller, extending precise and fast temperature control of the section with an uncertainty of ±0.01  K.^37^^–^^39^ Before measurement, the calibration of the refractometer was performed using double distilled water.^38^ After placing the section on the sample holder, a magnetic cover was placed on the prism to cover it entirely to ensure better light–tissue contact without external stray light. To assess the device sensitivity and precision, the RI measurement of each tissue section was repeated three times, and the average of these three measurements was used to determine the RI of each section. All measurements were conducted at room temperature (around 22°C).

### 2.4 Collagen Orientation Measurement

The sections extracted from specimen B (Fig. 1) were subjected to additional analysis to determine the orientation of the collagen fibers using PLM. PLM imaging was performed using an Abrio PLM system (CRi, Inc., Woburn, Massachusetts, United States) staged on a conventional light microscope (Nikon Diaphot TMD, Nikon, Inc., Shinagawa, Tokyo, Japan).^40^^,^^41^ The Abrio PLM system consisted of a circular polarizer, a green bandpass filter, and a computer-controlled analyzer consisting of two liquid crystal polarizers and a charge-coupled device camera. All samples were imaged in uniform orientation with a 4.0× objective, which resulted in a pixel size of 2.53×2.53  μm.^2^ In the orientation images, 0 and 90 deg correspond to the orientations parallel and perpendicular to the cartilage surface, respectively.^42^

## 3 Statistical Analysis

To assess the relationship between the biomechanical properties of human articular cartilage and its depth-wise RI, the extracted sections from all samples were grouped based on the zonal segmentation across the tissue depth, e.g., the SZ, MZ, and DZ. The normality of the RI datasets was also checked prior to analysis, using the Anderson–Darling test. The normality test was performed on each group of the RI, PLM, and biomechanical properties datasets, and based on the outcome, the non-parametric Kruskal–Wallis test was used for the non-normal dataset. Similarly, Spearman’s and Pearson’s correlation coefficients were employed for non-normal and normal datasets, respectively, to determine the correlation between the depth-wise RI and biomechanical properties of the cartilage samples as a function of collagen fibril orientation.

In the statistical tests, a p−value lower than 0.05 was considered to reflect statistical significance. All statistical analyses were performed using MATLAB (MathWorks, Natick, Massachusetts, United States, version R2019b).

## 4 Results

A positive correlation with statistically significant relations (p−values<0.05) was observed between the RI and the biomechanical properties (EM, IM, and DM) along the tissue depth for each zone (Table 1). Likewise, a lower positive correlation with statistically significant relations (p−values<0.05) was also observed for collagen fibril orientation of all zones with the biomechanical properties (Table 2). The empty cells for Spearman’s and Pearson’s correlation reflect that the datasets of the variables concerned are non-normal and normal, respectively.

**Table 1:** Summary of correlations between the RI and cartilage biomechanical properties in the SZs, MZs, and DZs of the articular cartilage.

	Pearson coefficient (R)	Spearman’s correlation (R)	p−value
SZRI-EM	—	0.4385	$7.1\times 10^{-5}$
SZRI-IM	—	0.2796	$6.1\times 10^{-4}$
SZRI-DM	—	0.3003	$5.3\times 10^{-3}$
MZRI-EM	0.0615	—	$1.4\times 10^{-7}$
MZRI-IM	0.5107	—	$3.7\times 10^{-4}$
MZRI-DM	0.4803	—	$2.1\times 10^{-4}$
DZRI-EM	—	0.3882	$3.1\times 10^{-4}$
DZRI-IM	0.6152	—	0.0018
DZRI-DM	0.5158	—	0.0027

**Table 2:** Summary of correlation between the collagen fibril orientation angle and cartilage biomechanical properties in the SZs, MZs, and DZs of articular cartilage.

	Pearson coefficient (R)	Spearman’s correlation (R)	p−value
SZPLM-EM	—	0.6738	$3.2\times 10^{-7}$
SZPLM-IM	−0.3063	—	$1.8\times 10^{-7}$
SZPLM-DM	0.0938	—	$6.8\times 10^{-6}$
MZPLM-EM	—	−0.0802	$6.7\times 10^{-8}$
MZPLM-IM	—	−0.2250	$7.8\times 10^{-8}$
MZPLM-DM	—	0.0506	$6.6\times 10^{-8}$
DZPLM-EM	—	0.4205	$1.5\times 10^{-6}$
DZPLM-IM	0.5220	—	$4.3\times 10^{-5}$
DZPLM-DM	0.2729	—	$1.2\times 10^{-3}$

### 4.1 Mapping of Zonal Refractive Index, Biomechanical Properties (EM, IM, and DM) with Collagen Fibril Orientation of Human Articular Cartilage

**Fig. 2:**
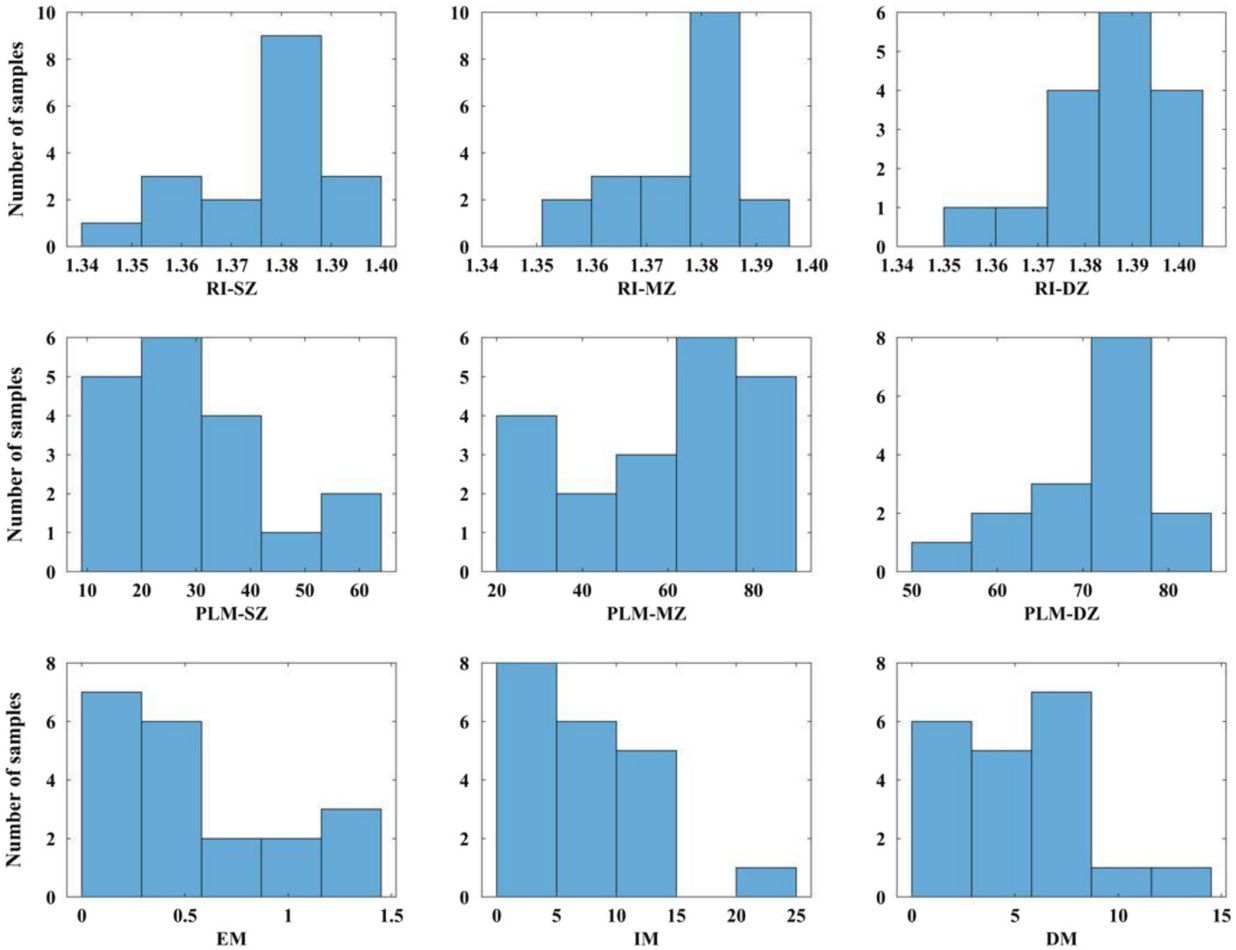
Zonal histograms for the RI, collagen fibril orientation (PLM), and histogramic illustration for distribution of biomechanical properties (EM, IM, and DM) of human articular cartilage.

**Fig. 3:**
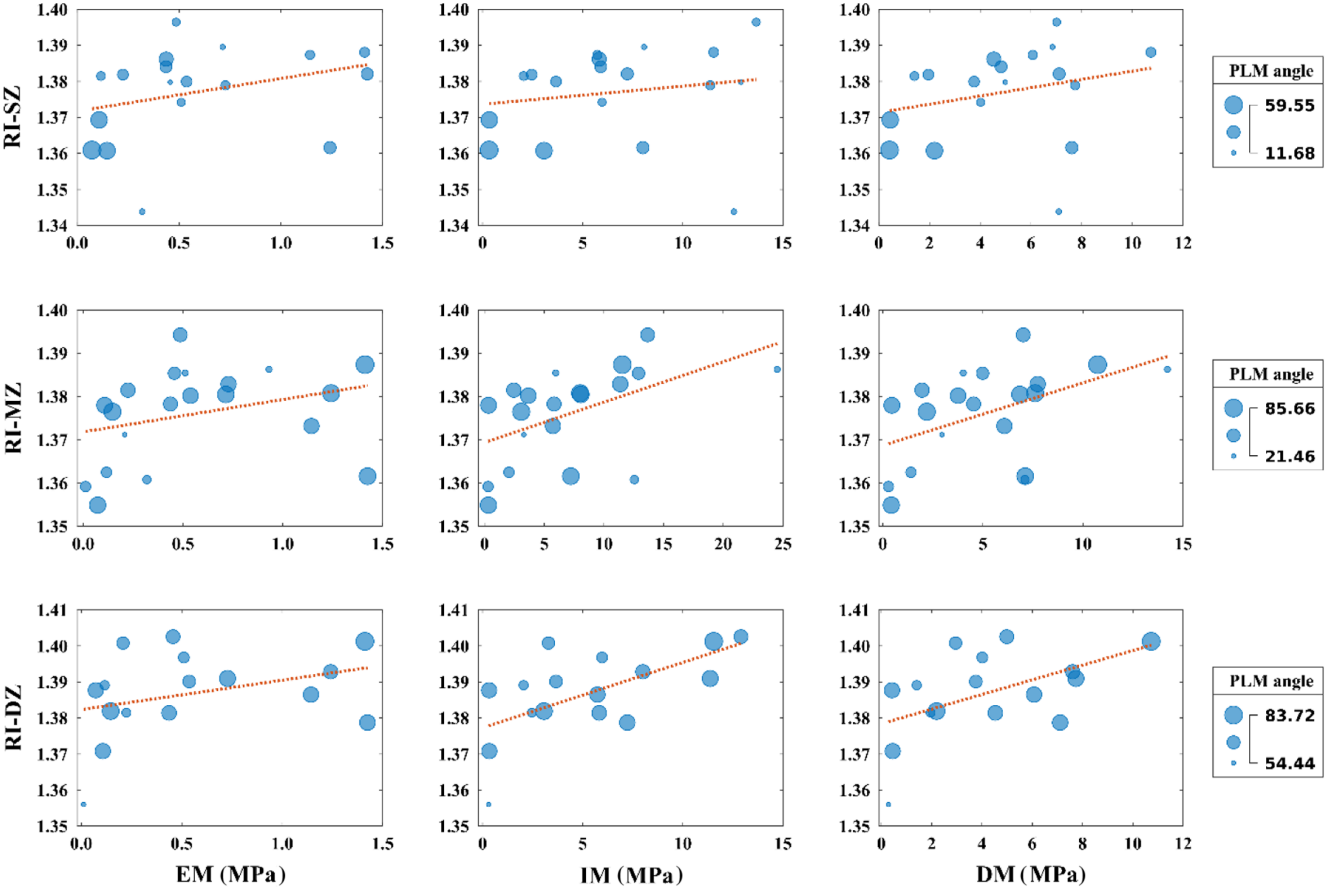
Mapping of the zonal RI and biomechanical properties (EM, IM, and DM) with collagen fibril orientation [PLM angle (deg)] of human articular cartilage.

Depth-wise distribution of the RI, collagen fibril orientation, and biomechanical properties (EM, IM, and DM) is illustrated in the histograms in Fig. 2. The collagen fibril orientation shows a consistent depth-dependent variation across the tissue depth as shown in Fig. 3. The collagen fibril orientation for the SZ, MZ, and DZ shows variation between 11.7 and 59.6 deg, 21.5 and 85.7 deg, and 54.4 and 83.7 deg, respectively.

In addition, the relationship between the zonal RI and biomechanical properties (IM, EM, and DM) shows an increasing trend for the SZ, MZ, and DZ. A similar trend can be observed with the collagen fibril orientation as illustrated in Fig. 3.

Statistically significant differences were observed between the RI of the SZ and EM, IM, and DM, with Spearman’s correlation of 0.4385, 0.2796, and 0.3003, respectively, and p<0.001 in all cases, as illustrated in Table 1. Similarly, statistically significant differences were observed between the RI of the MZ and EM, IM, and DM, with Pearson’s coefficient of 0.0615, 0.5107, and 0.4803, respectively, and p<0.001 in all cases (Table 1). Likewise, statistically significant differences were noticed between the RI of the DZ and EM, IM, and DM, with Spearman’s correlation of 0.3882 for EM and Pearson coefficient of 0.6152 and 0.5158 for IM and DM, respectively, and p<0.001 in all cases as illustrated in Table 1.

Similar to RI, statistically significant correlations were observed between collagen fibril orientation in the SZ and EM, IM, and DM, with Spearman’s correlation of 0.6738 for EM and Pearson coefficient of −0.3063 and 0.0938 for IM and DM, respectively, with p<0.001 in all cases (Table 2). Similarly, statistically significant correlations were also noticed between collagen fibril orientation in the MZ and EM, IM, and DM, with Spearman’s correlation of −0.0802, −0.2250, and 0.0506, respectively, and p<0.001 in all cases (Table 2). Finally, statistically significant correlations were observed between collagen fibril orientation in the DZ with EM, IM, and DM, having Spearman’s correlation = 0.4205 with EM and Pearson coefficients = 0.5220, and 0.2729, well within the range reported,^43^ with IM and DM, respectively, and p<0.001 in all cases as illustrated in Table 2.

## 5 Discussion

In this study, we investigated the relationship between the depth-wise RI and mechanical properties of human AC. Although the potential of RI for the characterization of articular cartilage integrity has been previously demonstrated,^1^^,^^44^ no study, to the best of our knowledge, has investigated the relationship between the depth-wise RI and the biomechanical properties of human AC. As the propagation of light in biological tissues is impacted by the ECM constituents^45^ and cartilage structure and composition are known to vary along its depth, we hypothesized that there would be a depth-dependent relationship between the cartilage RI and its mechanical characteristics.^46^

The observed variations in the relationship between the RI and biomechanical properties of human AC are probably related to depth-wise variation in the tissue structure, composition, and integrity. For example, collagen fibril orientation in the SZ becomes less parallel to the tissue surface in the early stages of degeneration in comparison to healthy tissue.^47^ This potentially leads to alteration of the RI as a function of tissue depth and degeneration.^27^

The knowledge of the depth-wise variation of RI and in relation to tissue properties and degenerative state is important for developing models for understanding the propagation of light in biological tissues, such as Monte Carlo simulation—a gold standard method for investigating the interaction of light in biological tissue.^48^ Thus, detailed knowledge of the variation RI with respect to the structure and composition of articular cartilage along its depth could provide critical information for accurately modeling the interaction of light in the tissue.

The relationship between the RI and the biomechanical properties of AC is likely due to an indirect relationship with its collagen fiber network.^31^ The collagen fibril network is known to have a major effect on cartilage mechanical properties, especially under dynamic and instantaneous loading.^19^^,^^49^ Studies have shown that the variation in the fibrillar architecture of different cartilage zones affects its mechanical properties.^49^^,^^50^ Furthermore, given its amount (22% of ECM’s wet weight), varying depth-wise orientation, and fibril diameter, collagen is the major light-scattering component of the articular cartilage.^51^^,^^52^ Although chondrocytes may also contribute to the scattering of light in the cartilage, their effect is likely to be minimal due to their dispersed nature and relatively low concentration (2% of volume) in ECM.

The relationship between the RI and biomechanical properties of human AC provides an indication of the potential of the RI for diagnostic characterization of cartilage properties. This relationship is valuable as it can be further developed into an approach for non-destructively estimating the biomechanical properties of articular cartilage, which are important biomarkers of articular cartilage degeneration. For example, cartilage integrity based on the OARSI score has been shown to be related to the biomechanical properties of the tissue.^53^ Likewise, an increase in the OARSI score has been reported to be related to the decrease in the elastic (equilibrium/dynamic/IM) and increase in the viscous (phase difference) properties of the cartilage.^21^ Furthermore, it has been shown that healthy cartilage has significantly higher EM compared with that of early and advanced OA cartilages.^20^^,^^54^ Thus, the RI could provide an indicator for estimating articular cartilage integrity through the relation between the tissue’s mechanical properties and its integrity score, evaluated via OARSI scoring.

Indentation testing has shown a good capability to reveal alterations in tissue properties in the SZ of cartilage, where the early signs of OA can be observed^47^^,^^55^ or even initiated during traumatic joint injury (post-traumatic OA). This is often characterized by fibrillation of the superficial collagen network (one of the earliest signs of OA^56^), further supporting the use of an indentation test in this study. Likewise, earlier studies have demonstrated the loss of PGs as a potential indicator for assessing the development of OA.^57^^–^^59^ A decrease in cartilage PG content decreases its swelling pressure, resulting in increased permeability and alteration of the load-bearing ability of its matrix.^60^ Thus, upon PG content depletion, the permeability of the ECM would increase significantly.^61^^,^^62^ Consequently, a recent study^63^ has reported that changes in permeability result in altered mechanical properties, leading to cartilage matrix damage and eventually OA and alteration of collagen integrity of the tissue.^19^

Thus, the relationship between RI and biomechanical properties of articular cartilage could potentially enable the development of optical diagnostic methods that could enable the detection of early degenerative changes in the cartilage.^64^^,^^65^

Likewise, in accordance with the structure–function relation, the RI and the biomechanical response of biological tissues are intrinsically related to the tissue’s structure and composition and thus could be a potential biomarker of tissue integrity. For example, the RI could serve as a useful indicator in cartilage tissue engineering for monitoring the deposition of cartilage ECM during tissue growth in a bioreactor. There are currently no studies in the literature, to the best of our knowledge, on the relationship between depth-wise RI and biomechanical properties of human articular cartilage with respect to potential *in vivo* application.

Taken together, the estimation of cartilage integrity via the prediction of the biomechanical properties from the RI (i.e., based on the relationship between OARSI scores and biomechanical properties of articular cartilage) can be potential applications of this study in the future. The main limitation of this study is the relatively low number of samples extracted from the knees of three cadavers. This limited our ability to draw a firm conclusion; thus, more detailed future investigations with a greater number of samples are needed. Samples extracted from cadavers of varying ages and from joints of varying degenerative states would allow for a better assessment of the relationship between the depth-wise RI and mechanical properties of human AC. The other key limitation of this study was the removal of the initial first few tens of micrometers from the articular surface. This was done to flatten the naturally curved articular surface to allow the preparation of uniformly flat sections. We believe that this did not significantly affect the estimated RI of the SZ in the case of healthy human articular cartilage samples; however, this may not be the case for pathological samples as it may result in the loss of some useful details in the SZ.

## 6 Conclusion

In conclusion, the present results indicate that there is a correlation between the RI and depth-wise variation of biomechanical properties of human articular cartilage. This finding is consistent with the depth-wise variation in cartilage composition and structure and provides critical information that could be useful for accurate modeling of light–tissue interaction using methods such as Monte Carlo simulation. For example, accounting for the variation in the RI for the different zones of articular cartilage could enable the development of more accurate models of light propagation in cartilage as compared with using a single RI for the bulk tissue. Moreover, the outcome of this study could support the development of complex Monte Carlo simulation models that can take into account the depth-wise variation of the RI for forecasting cartilage degradation and progression of OA based on a prediction of the biomechanical properties.

## Data Availability

The concerned datasets and codes regarding the results presented in this paper can be provided upon request from the authors.

## References

[1] Wang K. et al., “Characterizing depth-dependent refractive index of articular cartilage subjected to mechanical wear or enzymic degeneration,” J. Biomed. Opt. 21(9), 095002 (2016). 10.1117/1.JBO.21.9.095002 27626900

[2] Khan B. et al., “Articular cartilage optical properties in the near-infrared (NIR) spectral range vary with depth and tissue integrity,” Biomed. Opt. Express 12(10), 6066–6080 (2021). 10.1364/BOE.430053 34745722 PMC8548021

[3] Buckwalter J. A. Mankin H. J., “Articular cartilage: part I,” J. Bone Jt. Surg. 79(4), 600 (1997). 10.2106/00004623-199704000-00021

[4] Chen S. S. et al., “Depth-dependent compressive properties of normal aged human femoral head articular cartilage: relationship to fixed charge density,” Osteoarthritis Cartilage 9(6), 561–569 (2001). 10.1053/joca.2001.0424 11520170

[5] Sophia Fox A. J. Bedi A. Rodeo S. A., “The basic science of articular cartilage: structure, composition, and function,” Sports Health 1(6), 461–468 (2009). 10.1177/1941738109350438 23015907 PMC3445147

[6] Klein T. J. et al., “Tissue engineering of articular cartilage with biomimetic zones,” Tissue Eng. Part B Rev. 15(2), 143–157 (2009). 10.1089/ten.teb.2008.0563 19203206 PMC3121783

[7] Patil S. G. et al., “Measurement of depth-dependence and anisotropy of ultrasound speed of bovine articular cartilage *in vitro*,” Ultrasound Med. Biol. 30(7), 953–963 (2004). 10.1016/j.ultrasmedbio.2004.04.009 15313327

[8] Jung E. et al., “Quantitative analysis of water distribution in human articular cartilage using terahertz time-domain spectroscopy,” Biomed Opt. Express 3(5), 1110–1115 (2012). 10.1364/BOE.3.001110 22567600 PMC3342186

[9] Kempson G. E. et al., “The tensile properties of the cartilage of human femoral condyles related to the content of collagen and glycosaminoglycans,” Biochim. Biophys. Acta 297(2), 456–472 (1973). 10.1016/0304-4165(73)90093-7 4267503

[10] Hunziker E. B. Quinn T. M. Häuselmann H.-J., “Quantitative structural organization of normal adult human articular cartilage,” Osteoarthritis Cartilage 10(7), 564–572 (2002). 10.1053/joca.2002.0814 12127837

[11] WHO Scientific Group on Rheumatic Diseases, Ed., Rheumatic Diseases: Report of a WHO Scientific Group, World Health Organization, Geneva (1992).

[12] Mansfield J. C. et al., “Collagen fiber arrangement in normal and diseased cartilage studied by polarization sensitive nonlinear microscopy,” J. Biomed. Opt. 13(4), 044020 (2008). 10.1117/1.2950318 19021348

[13] Wang Z. et al., “Tissue refractive index as marker of disease,” J. Biomed. Opt. 16(11), 116017 (2011). 10.1117/1.3656732 22112122 PMC3223513

[14] Tuchin V. V., “Polarized light interaction with tissues,” J. Biomed. Opt. 21(7), 071114 (2016). 10.1117/1.JBO.21.7.071114 27121763

[15] Schmitt J. M. Kumar G., “Optical scattering properties of soft tissue: a discrete particle model,” Appl. Opt. 37(13), 2788–2797 (1998). 10.1364/AO.37.002788 18273225

[16] Tsenova V. Stoykova E. V., “Refractive index measurement in human tissue samples,” Proc. SPIE 5226, 413–417 (2003). 10.1117/12.519584

[17] Wang C. C.-B. et al., “Optical determination of anisotropic material properties of bovine articular cartilage in compression,” J. Biomech. 36(3), 339–353 (2003). 10.1016/S0021-9290(02)00417-7 12594982 PMC2809654

[18] Ebert D. W. et al., “Articular cartilage optical properties in the spectral range 300--850 nm,” J. Biomed. Opt. 3(3), 326–333 (1998). 10.1117/1.429893 23015086

[19] Ebrahimi M. et al., “Elastic, viscoelastic and fibril-reinforced poroelastic material properties of healthy and osteoarthritic human tibial cartilage,” Ann. Biomed. Eng. 47(4), 953–966 (2019). 10.1007/s10439-019-02213-4 30690688 PMC8494710

[20] Julkunen P. et al., “Stress–relaxation of human patellar articular cartilage in unconfined compression: prediction of mechanical response by tissue composition and structure,” J. Biomech. 41(9), 1978–1986 (2008). 10.1016/j.jbiomech.2008.03.026 18490021

[21] Ebrahimi M. et al., “Elastic, dynamic viscoelastic and model-derived fibril-reinforced poroelastic mechanical properties of normal and osteoarthritic human femoral condyle cartilage,” Ann. Biomed. Eng. 49(9), 2622–2634 (2021). 10.1007/s10439-021-02838-4 34341898 PMC8455392

[22] Lamela M. J. et al., “Dynamic compressive properties of articular cartilages in the porcine temporomandibular joint,” J. Mech. Behavior Biomed. Mater. 23, 62–70 (2013). 10.1016/j.jmbbm.2013.04.006 23660305

[23] Julkunen P. et al., “Mechanical characterization of articular cartilage by combining magnetic resonance imaging and finite-element analysis—a potential functional imaging technique,” Phys. Med. Biol. 53(9), 2425 (2008). 10.1088/0031-9155/53/9/014 18421123

[24] Desrochers J. Amrein M. A. Matyas J. R., “Structural and functional changes of the articular surface in a post-traumatic model of early osteoarthritis measured by atomic force microscopy,” J. Biomech. 43(16), 3091–3098 (2010). 10.1016/j.jbiomech.2010.08.009 20817164

[r25] Goldring S. R. Goldring M. B., “Changes in the osteochondral unit during osteoarthritis: structure, function and cartilage–bone crosstalk,” Nat. Rev. Rheumatol. 12(11), 632–644 (2016). 10.1038/nrrheum.2016.148 27652499

[26] Knecht S. Vanwanseele B. Stüssi E., “A review on the mechanical quality of articular cartilage—implications for the diagnosis of osteoarthritis,” Clin. Biomech. 21(10), 999–1012 (2006). 10.1016/j.clinbiomech.2006.07.001 16979270

[27] Khan B. et al., “Refractive index of human articular cartilage varies with tissue structure and composition,” J. Opt. Soc. Am. A 40(12), 2205–2214 (2023). 10.1364/JOSAA.498722 38086029

[28] Jurvelin J. S. et al., “Comparison of optical, needle probe and ultrasonic techniques for the measurement of articular cartilage thickness,” J. Biomech. 28(2), 231–235 (1995). 10.1016/0021-9290(94)00060-H 7896866

[29] Qu C. et al., “Effects of freeze-thaw cycle with and without proteolysis inhibitors and cryopreservant on the biochemical and biomechanical properties of articular cartilage,” Cartilage 5(2), 97–106 (2014). 10.1177/1947603513515998 26069689 PMC4297078

[30] Király K. et al., “Application of selected cationic dyes for the semiquantitative estimation of glycosaminoglycans in histological sections of articular cartilage by microspectrophotometry,” Histochem. J. 28(8), 577–590 (1996). 10.1007/BF02331378 8894661

[31] Kafian-Attari I. et al., “Tissue optical properties combined with machine learning enables estimation of articular cartilage composition and functional integrity,” Biomed. Opt. Express 11(11), 6480–6494 (2020). 10.1364/BOE.402929 33282503 PMC7687936

[32] Prakash M. et al., “Near-infrared spectroscopy enables quantitative evaluation of human cartilage biomechanical properties during arthroscopy,” Osteoarthritis Cartilage 27(8), 1235–1243 (2019). 10.1016/j.joca.2019.04.008 31026649

[33] Korhonen R. K. et al., “Comparison of the equilibrium response of articular cartilage in unconfined compression, confined compression and indentation,” J. Biomech. 35(7), 903–909 (2002). 10.1016/S0021-9290(02)00052-0 12052392

[34] Kiviranta P. et al., “Collagen network primarily controls Poisson’s ratio of bovine articular cartilage in compression,” J. Orthop. Res. 24(4), 690–699 (2006). 10.1002/jor.20107 16514661

[35] Jurvelin J. S. Buschmann M. D. Hunziker E. B., “Optical and mechanical determination of Poisson’s ratio of adult bovine humeral articular cartilage,” J. Biomech. 30(3), 235–241 (1997). 10.1016/S0021-9290(96)00133-9 9119822

[36] Hayes W. C. et al., “A mathematical analysis for indentation tests of articular cartilage,” J. Biomech. 5(5), 541–551 (1972). 10.1016/0021-9290(72)90010-3 4667277

[37] Bhatia S. C. Tripathi N. Dubey G. P., “Refractive indices of binary liquid mixtures of (decane + benzene) and (hexadecane + benzene, or + hexane) at various temperatures,” Indian J. Chem. 41(2), 266–269 (2002).

[r38] Mohammadi M. D. Hamzehloo M., “Densities, viscosities, and refractive indices of binary and ternary mixtures of methanol, acetone, and chloroform at temperatures from (298.15–318.15) K and ambient pressure,” Fluid Phase Equilib. 483, 14–30 (2019). 10.1016/j.fluid.2018.10.024

[39] Pandey V. et al., “Refractive indices and their related properties for binary mixtures containing 2-diethylethanolamine with 1-propanol and 1-butanol,” J. Solution Chem. 49, 1459–1472 (2020). 10.1007/s10953-020-01032-9

[40] Hänninen N. et al., “Orientation anisotropy of quantitative MRI relaxation parameters in ordered tissue,” Sci. Rep. 7(1), 9606 (2017). 10.1038/s41598-017-10053-2 28852032 PMC5574987

[41] Rieppo J. et al., “Practical considerations in the use of polarized light microscopy in the analysis of the collagen network in articular cartilage,” Microsc. Res. Tech. 71(4), 279–287 (2008). 10.1002/jemt.20551 18072283

[42] Sarin J. K. et al., “Combination of optical coherence tomography and near infrared spectroscopy enhances determination of articular cartilage composition and structure 1,” Sci. Rep. 7(1), 10586 (2017). 10.1038/s41598-017-10973-z 28878384 PMC5587743

[43] Hauke J. Kossowski T., “Comparison of values of Pearson’s and Spearman’s correlation coefficients on the same sets of data,” Quaest. Geographicae 30(2), 87–93 (2011). 10.2478/v10117-011-0021-1

[44] Wang S.-Z. et al., “Assessment of depth and degeneration dependences of articular cartilage refractive index using optical coherence tomography in vitro,” Connect. Tissue Res. 51(1), 36–47 (2010). 10.3109/03008200902890161 20067415

[45] Jacques S. L, “Optical properties of biological tissues: a review,” Phys. Med. Biol. 58(11), 37–61 (2013). 10.1088/0031-9155/58/11/R37 23666068

[46] Tuchin V. V., “Tissue optics and photonics: light-tissue interaction 2,” J. Biomed. Photonics Eng. 1(2), 98–134 (2015). 10.18287/JBPE-2015-1-2-98

[47] Saarakkala S. et al., “Depth-wise progression of osteoarthritis in human articular cartilage: investigation of composition, structure and biomechanics,” Osteoarthritis Cartilage 18(1), 73–81 (2010). 10.1016/j.joca.2009.08.003 19733642

[48] Wang L. Jacques S. L., “Monte Carlo modeling of light transport in multi-layered tissues in standard C,” The University of Texas, MD Anderson Cancer Center, Houston 4(11) (1992).

[49] Soltz M. A. Ateshian G. A., “Interstitial fluid pressurization during confined compression cyclical loading of articular cartilage,” Ann. Biomed. Eng. 28(2), 150–159 (2000). 10.1114/1.239 10710186

[50] Eyre D., “Articular cartilage and changes in arthritis: collagen of articular cartilage,” Arthritis Res. Ther. 4(1), 30 (2001). 10.1186/ar380 PMC128915 11879535

[51] Hargrave-Thomas E. J. et al., “The bovine patella as a model of early osteoarthritis,” J. Anatomy 223(6), 651–664 (2013). 10.1111/joa.12115 PMC3842206 24111904

[52] Poole J. J. A. Mostaço-Guidolin L. B., “Optical microscopy and the extracellular matrix structure: a review,” Cells 10(7), 1760 (2021). 10.3390/cells10071760 34359929 PMC8308089

[53] Waldstein W. et al., “OARSI osteoarthritis cartilage histopathology assessment system: a biomechanical evaluation in the human knee,” J. Orthop. Res. 34(1), 135–140 (2016). 10.1002/jor.23010 26250350

[54] Julkunen P. et al., “Characterization of articular cartilage by combining microscopic analysis with a fibril-reinforced finite-element model,” J. Biomech. 40(8), 1862–1870 (2007). 10.1016/j.jbiomech.2006.07.026 17052722

[55] Guilak F. et al., “Mechanical and biochemical changes in the superficial zone of articular cartilage in canine experimental osteoarthritis,” J. Orthop. Res. 12(4), 474–484 (1994). 10.1002/jor.1100120404 8064478

[56] Changoor A. et al., “Structural characteristics of the collagen network in human normal, degraded and repair articular cartilages observed in polarized light and scanning electron microscopies,” Osteoarthritis and Cartilage 19(12), 1458–1468 (2011). 10.1016/j.joca.2011.09.007 22015933

[57] Harkey M. S. et al., “Osteoarthritis-related biomarkers following anterior cruciate ligament injury and reconstruction: a systematic review,” Osteoarthritis Cartilage 23(1), 1–12 (2015). 10.1016/j.joca.2014.09.004 25219671

[r58] Inerot S. et al., “Articular-cartilage proteoglycans in aging and osteoarthritis,” Biochem. J. 169(1), 143–156 (1978). 10.1042/bj1690143 629741 PMC1184203

[59] Appleyard R. C. et al., “Topographical analysis of the structural, biochemical and dynamic biomechanical properties of cartilage in an ovine model of osteoarthritis,” Osteoarthritis Cartilage 11(1), 65–77 (2003). 10.1053/joca.2002.0867 12505489

[60] Muir H., “Proteoglycans of cartilage,” J. Clin. Pathol. 31(Suppl 12), 67–81 (1978). 10.1136/jcp.31.Suppl_12.67 PMC1347125 365895

[61] Wang Q. et al., “Altered osmotic swelling behavior of proteoglycan-depleted bovine articular cartilage using high frequency ultrasound,” Phys. Med. Biol. 53(10), 2537 (2008). 10.1088/0031-9155/53/10/006 18424876

[62] Lai W. M. Hou J. S. Mow V. C., “A triphasic theory for the swelling and deformation behaviors of articular cartilage,” J. Biomech. Eng. 113(3), 245–258 (1991). 10.1115/1.2894880 1921350

[63] Mäkelä J. T. A. et al., “Very early osteoarthritis changes sensitively fluid flow properties of articular cartilage,” J. Biomech. 48(12), 3369–3376 (2015). 10.1016/j.jbiomech.2015.06.010 26159056

[64] Pollard T. C. B. Gwilym S. E. Carr A. J., “The assessment of early osteoarthritis,” J. Bone Jt. Surg. Br. 90(4), 411–421 (2008). 10.1302/0301-620X.90B4.20284 18378911

[65] Yin J. Xia Y., “Proteoglycan concentrations in healthy and diseased articular cartilage by Fourier transform infrared imaging and principal component regression,” Spectrochim. Acta A Mol. Biomol. Spectrosc. 133, 825–830 (2014). 10.1016/j.saa.2014.05.092 25000570 PMC4133143

